# Supplementary material for: Topological quadratic-node semimetal in a photonic microring lattice
Source: Nat Commun. 2023 Jun 2;14:3206. doi: 10.1038/s41467-023-38861-3 (PMC10238381; doi:10.1038/s41467-023-38861-3)
Supplement: Supplementary file 1 — Supplementary Information [file 41467_2023_38861_MOESM1_ESM.pdf]

# Supplementary Information for
# Topological quadratic-node semimetal in a photonic microring lattice

Zihe Gao,[1*] Haoqi Zhao,[2] Tianwei Wu,[1] Xilin Feng,[2] Zhifeng Zhang,[1,2] Xingdu Qiao,[2] Ching-Kai Chiu,[3*] Liang Feng[1,2*]

[1]*Department of Materials Science and Engineering, University of Pennsylvania, Philadelphia, PA 19104, USA*

[2]*Department of Electrical and Systems Engineering, University of Pennsylvania, Philadelphia, PA 19104, USA*

[3]*RIKEN Interdisciplinary Theoretical and Mathematical Sciences (iTHEMS), Wako, Saitama 351-0198, Japan*

*Corresponding author. Email: zihegao@seas.upenn.edu (Z.G.); ching-kai.chiu@riken.jp (C.-K.C.); fenglia@seas.upenn.edu (L.F.)

## 1. Rotation operator and coupling matrix in photonic microring lattice

Both the rotation operator and the coupling matrix between adjacent rings in the tight-binding model depend on the whispering gallery mode (WGM) order in the microring resonator. Denote an arbitrary location along the ring by its angular location $\Phi \in [0,2\pi)$, the counterclockwise (CCW) and clockwise (CW) traveling WGMs have their electromagnetic field wind along the ring as $\propto e^{\pm iN\Phi}$ [1], where $N$ is the WGM mode order (see Supplementary Figure 1a). We further define (using 2D TM mode as an example) if the modes have phase of $\mathrm{Arg}(H_z) = 0$ at $\Phi = 0$, the CCW and CW traveling modes are written as $(1,0)^T$ and $(0,1)^T$ in the pseudospin representation, respectively (i.e., setting the phase origin to be at $\Phi = 0$). The rotation operator rotates the wavefunction by angle $\beta$. An arbitrary CCW mode is rotated from $(a,0)^T$ to $\left(ae^{-iN\beta},0\right)^T$ and the CW mode from $(0,b)^T$ to $\left(0,be^{iN\beta}\right)^T$, where $a$ and $b$ are arbitrary complex numbers. Equivalently this rotation is represented by $C_\beta\psi$, where $C_\beta = \begin{pmatrix} e^{-iN\beta} & 0 \\ 0 & e^{iN\beta} \end{pmatrix}$. The rotation of Hamiltonian follows $C_\beta H C_\beta^{-1}$.

When we consider the coupling coefficients in the coupling matrix, if the WGM order is $N = 6n$ where $n \in \mathbb{N}$, for all the six locations where coupling occurs the phase is zero and this scenario should reduce to the simple case of evanescent coupling between two cavities with constant phase profiles. In this case the coupling coefficients are defined as 1 and the coupling matrix as $\sigma_x$. For example, in this case for a coupled 2-ring dimer the second-quantized Hamiltonian is $\hat{H} = -\sum \hat{C}_2^\dagger \kappa_0 \hat{C}_1 + h.c. = -(\hat{C}_2^\dagger \sigma_x \hat{C}_1 + \hat{C}_1^\dagger \sigma_x \hat{C}_2)$ where $\hat{C} = \begin{pmatrix} \hat{c}_{\circlearrowleft 1,2} \\ \hat{c}_{\circlearrowright 1,2} \end{pmatrix}$ is annihilation operator at ring 1 and 2, respectively. The first-quantized Hamiltonian matrix can then be written as a 4×4 matrix $H = -\begin{pmatrix} 0 & \sigma_x \\ \sigma_x & 0 \end{pmatrix}$ and it can be verified that the in-phase mode is the fundamental mode with lower energy. For any even-order WGMs, the coupling matrix in the horizontal direction (i.e., between two horizontally aligned rings) is $\kappa_0 = \sigma_x$ by our definition of

wavefunction phase, while for any odd-order WGMs, it is $\kappa_0 = -\sigma_x$. The coupling matrix along any other direction $\beta$ (in the triangular lattice $\beta \in \{0, \frac{\pi}{3}, \frac{2\pi}{3}, \pi, \frac{4\pi}{3}, \frac{5\pi}{3}\}$, see Supplementary Figure 1b) can be obtained using the rotation operator,

$$\kappa_\beta = C_\beta(\kappa_0) \equiv C_\beta \kappa_0 C_\beta^{-1} \tag{1}$$

Note that $C_\beta(\cdot)$ as a function is not to be confused with $C_\beta$ which is a matrix. For example, for even-order WGMs, the coupling matrix along $\beta$ direction would be

$$\kappa_\beta = C_\beta(\sigma_x) = \begin{pmatrix} 0 & e^{-2iN\beta} \\ e^{2iN\beta} & 0 \end{pmatrix} = \cos(2N\beta)\,\sigma_x + \sin(2N\beta)\,\sigma_y, \quad N = 2n \tag{2}$$

while for odd-order WGMs,

$$\kappa_\beta = C_\beta(-\sigma_x) = -\begin{pmatrix} 0 & e^{-2iN\beta} \\ e^{2iN\beta} & 0 \end{pmatrix} = -\cos(2N\beta)\,\sigma_x - \sin(2N\beta)\,\sigma_y, \quad N = 2n+1 \tag{3}$$

where $n \in \mathbb{N}$.

For mode order 34:

$$\begin{gathered} \kappa_0 = \sigma_x \\ \kappa_{\pi/3} = \begin{pmatrix} 0 & e^{-\frac{i2}{3}\pi} \\ e^{\frac{i2}{3}\pi} & 0 \end{pmatrix} = -\frac{1}{2}\sigma_x + \frac{\sqrt{3}}{2}\sigma_y \\ \kappa_{\frac{2\pi}{3}} = \begin{pmatrix} 0 & e^{\frac{i2}{3}\pi} \\ e^{-\frac{i2}{3}\pi} & 0 \end{pmatrix} = -\frac{1}{2}\sigma_x - \frac{\sqrt{3}}{2}\sigma_y \\ C_6 = \begin{pmatrix} e^{\frac{i2}{3}\pi} & 0 \\ 0 & e^{-\frac{i2}{3}\pi} \end{pmatrix}. \end{gathered} \tag{4}$$

Note that $C_6$ denotes $C_{\pi/3}$ .For mode order 35:

$$\begin{gathered} \kappa_0 = -\sigma_x, \\ \kappa_{\pi/3} = -\begin{pmatrix} 0 & e^{\frac{i2}{3}\pi} \\ e^{-\frac{i2}{3}\pi} & 0 \end{pmatrix} = \frac{1}{2}\sigma_x + \frac{\sqrt{3}}{2}\sigma_y \\ \kappa_{\frac{2\pi}{3}} = -\begin{pmatrix} 0 & e^{-\frac{i2}{3}\pi} \\ e^{\frac{i2}{3}\pi} & 0 \end{pmatrix} = \frac{1}{2}\sigma_x - \frac{\sqrt{3}}{2}\sigma_y \\ C_6 = \begin{pmatrix} e^{\frac{i\pi}{3}} & 0 \\ 0 & e^{-\frac{i\pi}{3}} \end{pmatrix}. \end{gathered} \tag{5}$$

It can be verified that when $N = 3n$, $\kappa_\beta$ is not winding (i.e., it has no dependence on $\beta$ when $\beta \in \{0, \frac{\pi}{3}, \frac{2\pi}{3}, \pi, \frac{4\pi}{3}, \frac{5\pi}{3}\}$). For $N = 34$ and $N = 35$, $\kappa_\beta$ winds in opposite directions. Moreover, along $k_y = 0$, the pseudospins in the band structure are flipped between $N = 34$ and $N = 35$, because of the extra minus sign in $\kappa_0$.

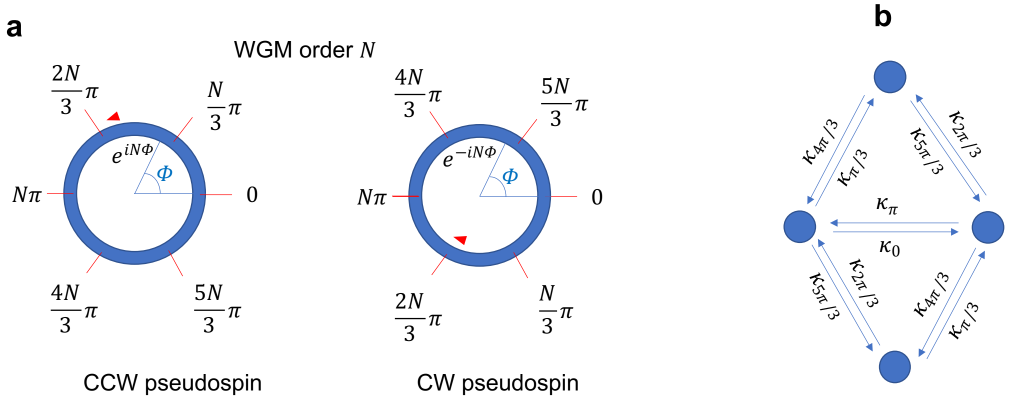


**Supplementary Figure 1. Phase profile of the WGM modes and the direction-dependent coupling matrix. a,** WGM phase winding along the ring for the CCW and CW pseudospins, respectively. **b,** Coupling matrices in a triangular lattice.

## 2. Time-domain tight-binding-model calculation of simultaneous Klein and anti-Klein tunneling

We study the triangular lattice with armchair termination in $x$ direction and infinite in $y$ direction (Supplementary Figure 2), a p-n junction box (the potential profile in $x$ direction gives us a p-n junction and the open boundaries put the p-n junction in a box, therefore we call this structure a p-n junction in a box). For a given $k_y$, after Fourier transform in y direction ($\hat{C}^\dagger_{x,y} = \frac{1}{N_y}\sum_{k_y} e^{ik_y y}\hat{C}^\dagger_{x,k_y}$), this lattice can be effectively described as a 1D chain with N atoms and lattice constant of $1/2$ :

$$\hat{H}(k_y) = \sum_x \hat{C}_x^\dagger V_x C_x - \hat{C}^\dagger_{x+1,k_y}\kappa_0\hat{C}_{x,k_y} - \hat{C}^\dagger_{x,k_y}\kappa_\pi\hat{C}_{x+1,k_y} - e^{ik_y\frac{\sqrt{3}}{2}}\hat{C}^\dagger_{x+\frac{1}{2},k_y}\kappa_{\pi/3}\hat{C}_{x,k_y} - e^{-ik_y\frac{\sqrt{3}}{2}}\hat{C}^\dagger_{x,k_y}\kappa_{4\pi/3}\hat{C}_{x+\frac{1}{2},k_y} - e^{ik_y\frac{\sqrt{3}}{2}}\hat{C}^\dagger_{x-\frac{1}{2},k_y}\kappa_{2\pi/3}\hat{C}_{x,k_y} - e^{-ik_y\frac{\sqrt{3}}{2}}\hat{C}^\dagger_{x,k_y}\kappa_{5\pi/3}\hat{C}_{x-\frac{1}{2},k_y}$$

$$= \begin{pmatrix}\hat{C}^\dagger_{0,k_y} & \hat{C}^\dagger_{\frac{1}{2},k_y} & \cdots & \hat{C}^\dagger_{\frac{N-1}{2},k_y}\end{pmatrix}\begin{pmatrix} V_0 & -t_{\mathrm{NN}} & -t_{\mathrm{NNN}} & 0 \\ -t'_{\mathrm{NN}} & V_{\frac{1}{2}} & -t_{\mathrm{NN}} & \ddots \\ -t'_{\mathrm{NNN}} & -t'_{\mathrm{NN}} & \ddots & \ddots \\ 0 & \ddots & \ddots & V_{\frac{N-1}{2}}\end{pmatrix}\begin{pmatrix}\hat{C}_{0,k_y} \\ \hat{C}_{\frac{1}{2},k_y} \\ \vdots \\ \hat{C}_{\frac{N-1}{2},k_y}\end{pmatrix} \tag{6}$$

where $t_{\mathrm{NN}} = e^{ik_y\frac{\sqrt{3}}{2}}\kappa_{2\pi/3} + e^{-ik_y\frac{\sqrt{3}}{2}}\kappa_{4\pi/3}$ , $t'_{\mathrm{NN}} = e^{ik_y\frac{\sqrt{3}}{2}}\kappa_{\pi/3} + e^{-ik_y\frac{\sqrt{3}}{2}}\kappa_{5\pi/3}$ , $t_{\mathrm{NNN}} = \kappa_\pi$ , $t'_{\mathrm{NNN}} = \kappa_0$ (NN and NNN denotes nearest-neighbor and next-nearest-neighbor), $V_x = v_x\sigma_x$ is the on-site potential at lattice coordinate $x$, and $\kappa_\beta$ is the coupling matrix in $\beta$ direction (see Supplementary Figure 1b). The matrix $H = \begin{pmatrix} V_0 & -t_{\mathrm{NN}} & -t_{\mathrm{NNN}} & 0 \\ -t'_{\mathrm{NN}} & V_{\frac{1}{2}} & -t_{\mathrm{NN}} & \ddots \\ -t'_{\mathrm{NNN}} & -t'_{\mathrm{NN}} & \ddots & \ddots \\ 0 & \ddots & \ddots & V_{\frac{N-1}{2}}\end{pmatrix}$ is the first-quantized Hamiltonian and we can calculate the wavefunction evolution according to the Schrödinger's equation (setting $\hbar = 1$)

$$\frac{d}{dt}\Psi = \frac{d}{dt}\begin{pmatrix} \psi_0 \\ \psi_{\frac{1}{2}} \\ \vdots \\ \psi_{\frac{N-1}{2}} \end{pmatrix} = -iH\Psi = -\mathrm{i}\begin{pmatrix} V_0 & -t_{\mathrm{NN}} & -t_{\mathrm{NNN}} & 0 \\ -t'_{\mathrm{NN}} & V_{\frac{1}{2}} & -t_{\mathrm{NN}} & \ddots \\ -t'_{\mathrm{NNN}} & -t'_{\mathrm{NN}} & \ddots & \ddots \\ 0 & \ddots & \ddots & V_{\frac{N-1}{2}} \end{pmatrix}\begin{pmatrix} \psi_0 \\ \psi_{\frac{1}{2}} \\ \vdots \\ \psi_{\frac{N-1}{2}} \end{pmatrix} \tag{7}$$

We construct a p-n junction with $N = 400$ atoms (total length of 200 with lattice constant of 1/2), where the left half is the n-region with $V = 0$, and the right half is the p-region with $V = 2$. The transition between n- and p-regions is set to be smooth (as a sigmoid function with width ~10) to suppress any inter-valley scattering (See Supplementary Figure 2b). We set the initial wavefunction to be a Gaussian function in k-space centered at $k_x = 0.4\pi$ ($k_x = -1.2\pi$) near the quadratic (Dirac) cone with energy ~1. In real space this initial wavefunction is a Gaussian pulse propagating from the n-region towards the p-region. Then we numerically calculate the evolution of this pulse using software package QuTip [2]. The pulse, depending on being a massive of massless chiral quasiparticle, show complete reflection or transmission upon incidence on the potential step, with 100% reflection (transmission) coefficient. In addition, intervalley scattering happens upon reflection at the left ($x = 0$) open boundary of the chain, and after that the wavefunction contains both Dirac- and quadratic-valley components and we can observe simultaneous Klein and anti-Klein tunneling. Note that upon reflection at the right boundary in the p-region, although the boundary can provide sufficient momentum for intervalley scattering, the spin conservation does not allow it and hence no intervalley scattering happens. Some snapshots through the evolution are captured in Supplementary Figure 3 and Supplementary Figure 4. The continuous evolution is recorded in the Supplementary Movie 4 and S5, with initial pulse in the quadratic- and Dirac-valley, respectively.

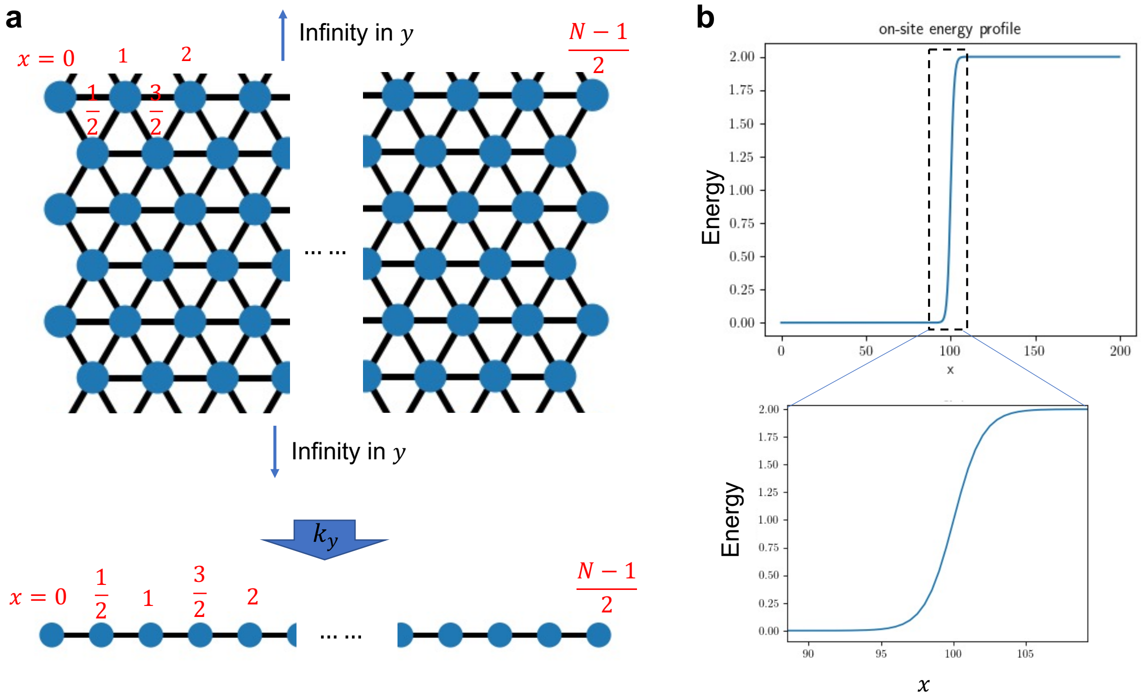


**Supplementary Figure 2. Tight-binding-model of a triangular lattice with potential profile in $x$ direction and infinite in $y$ direction, terminated with armchair open boundary. a,** The triangular lattice and the effective 1D chain for a given $k_y$. **b,** The potential profile for the p-n junctions.

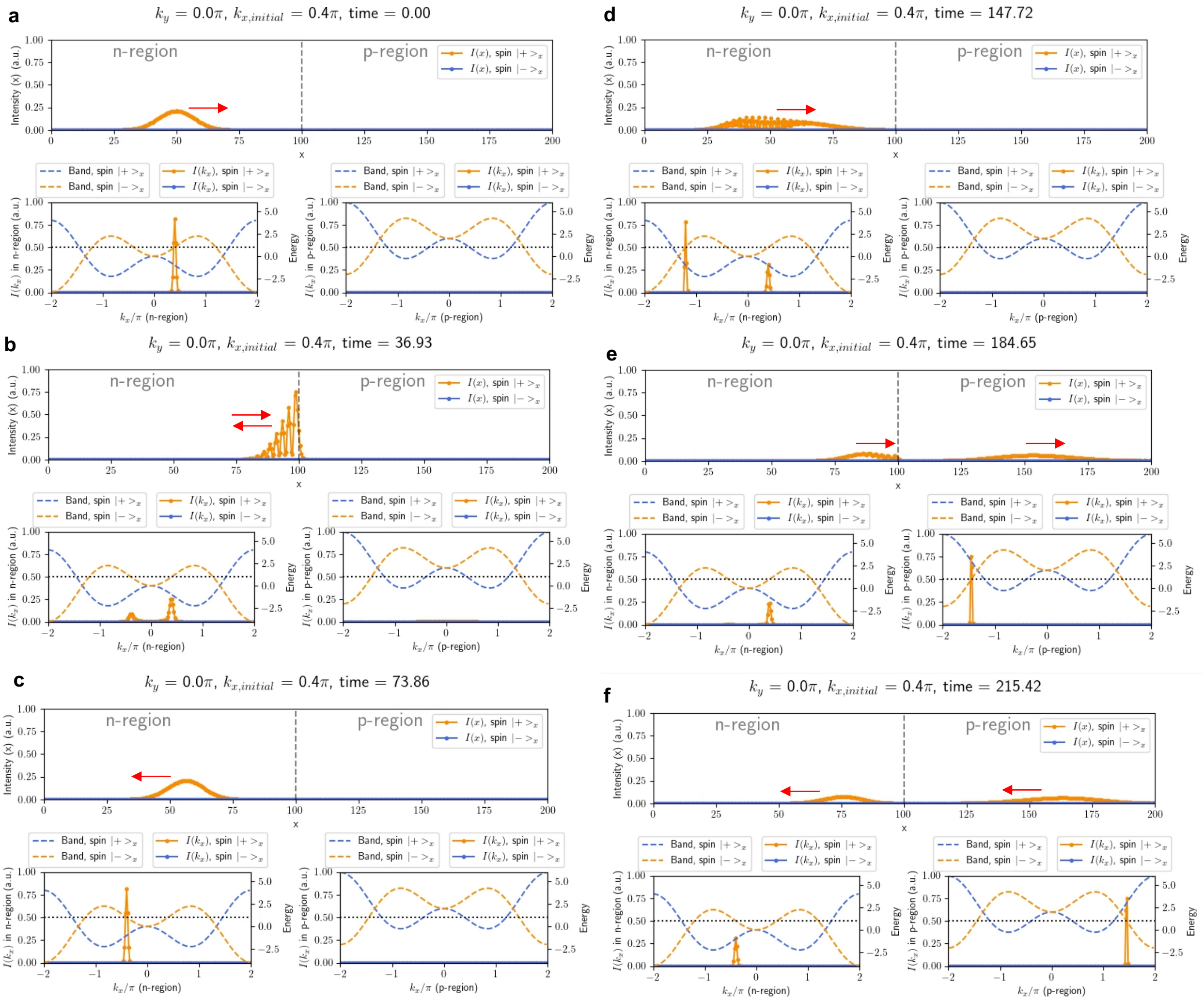


**Supplementary Figure 3. Evolution of the wavefunction in a p-n junction box, with initial pulse in the quadratic valley (see Supplementary Movie 4 for the continuous evolution).** For each time frame, the upper panel shows wavefunction intensity distribution in the real-space, and the two lower panels show the wavefunction intensity distribution in the momentum-space (solid lines) in the n- and p-regions respectively, overlayed with the band dispersion (dashed lines). Red arrows illustrate propagation direction of the pulse. The two spin components ($|+\rangle_x = (1,1)^T$ and $|-\rangle_x = (1,-1)^T$) are represented by orange and blue colors respectively. Throughout the evolution, spin is conserved and can be confirmed with the $-x$ spin intensity always being zero. **a,** The initial pulse of a massive chiral quasiparticle. **b,** Collision with the potential step. **c,** After total reflection (anti-Klein tunneling). **d,** Coexisting massive and massless chiral components after intervalley scattering at the left boundary. **e,** The massless chiral component completely transmitted into the p-region. **f,** The massive chiral component completely reflected back into the n-region.

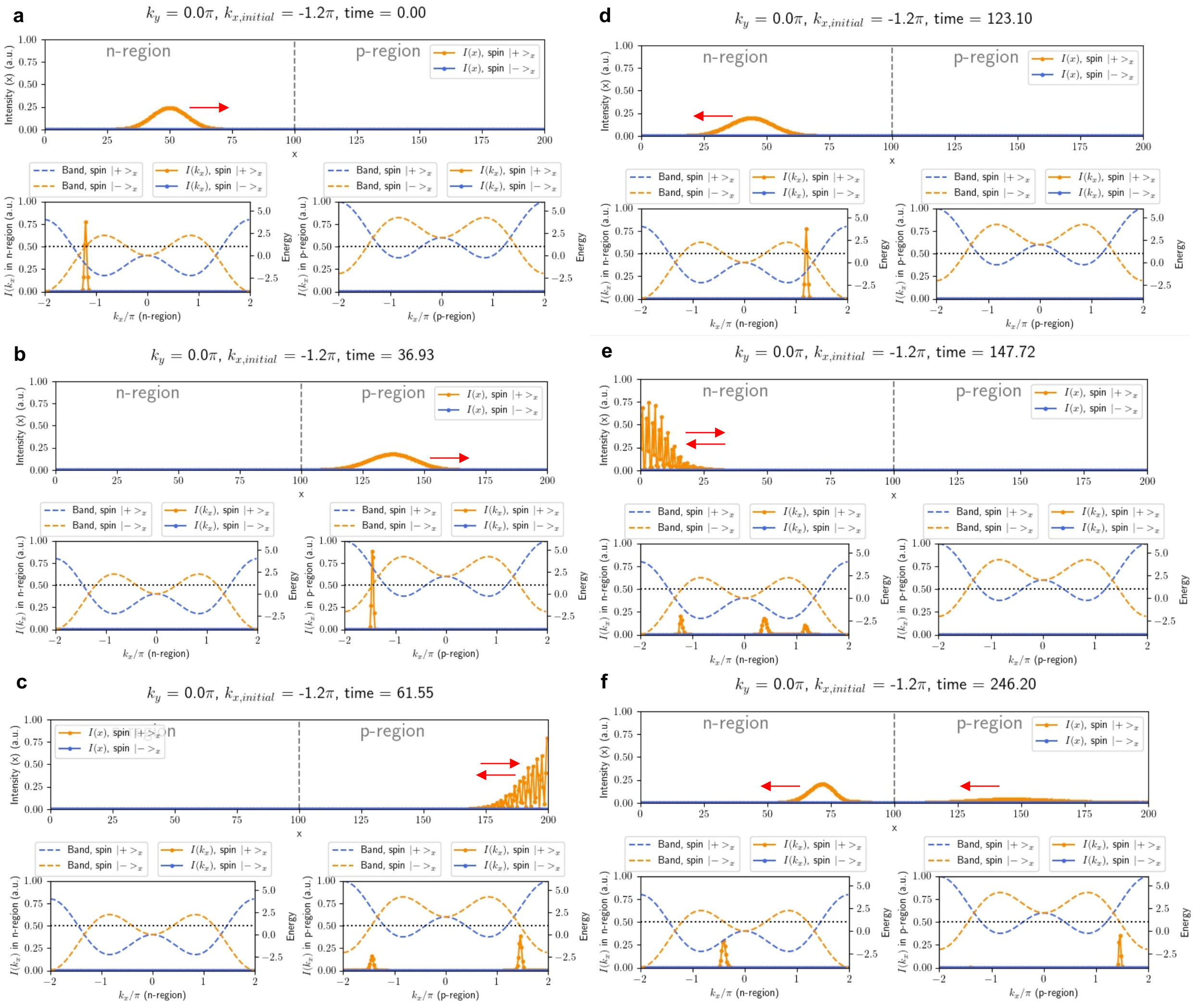


**Supplementary Figure 4. Evolution of the wavefunction in a p-n junction box, with initial pulse in the Dirac valley (see Supplementary Movie 5 for the continuous evolution).** For each time frame, the upper panel shows wavefunction intensity distribution in the real-space, and the two lower panels show the wavefunction intensity distribution in the momentum-space (solid lines) in the n- and p-regions respectively, overlayed with the band dispersion (dashed lines). Red arrows represent propagation direction of the pulse. The two spin components ($|+\rangle_x = (1,1)^T$ and $|-\rangle_x = (1,-1)^T$) are represented by orange and blue colors respectively. Though out the evolution, spin is conserved and can be confirmed with the $-x$ spin intensity always being zero. **a,** The initial pulse of a massless chiral quasiparticle. **b,** Complete transmission into the p-region (Klein tunneling). **c,** Collision at the right boundary (no intervalley scattering due to spin conservation). **d,** Complete transmission back to the n-region. **e,** Collision at the left boundary with intervalley scattering and coexisting massive and massless chiral components for a single particle. **f,** The massive chiral component completely reflected back into the n-region, while the massless chiral component continued into the p-region without reflection at the p-n junction.

## 3. Transmission and scattering in quadratic-node semimetal nanoribbon p-n junction

In the previous section we show the transport in an ideal p-n junction that is infinite in y direction. Here we study the transport in a p-n junction realized in a nanoribbon made of quadratic-node semimetal, which has finite width in y direction. The simulation is carried out using the software package Kwant [3]. Shown in

Supplementary Figure 5, the transport calculation is carried out in a system with a finite scattering region connected with two semi-infinite periodic ports (i.e., leads). The whole system under calculation is finite in $y$ direction, and hence represents a nanoribbon. The ports have translational symmetry in $x$ direction and their eigenmodes are labeled with the corresponding eigenvalues $k_x$. The transport in the p-n junction is then fully captured by the scattering matrix between input and output eigenmodes. In Figs. S6-S9, we show that in a nanoribbon, although the incident wave is not perfectly collimated (there is small but nonzero $k_y$), qualitatively we still observe the Klein tunneling and anti-Klein tunneling in the Dirac and quadratic valleys, respectively. The transmission (reflection) coefficients for Klein (anti-Klein) tunneling are as high as 96% (99%) for a nanoribbon width of 43, and remain $\gtrsim$ 90% even in a ribbon of less than 20 lattice constants in width and a sharp (step-function) potential barrier.

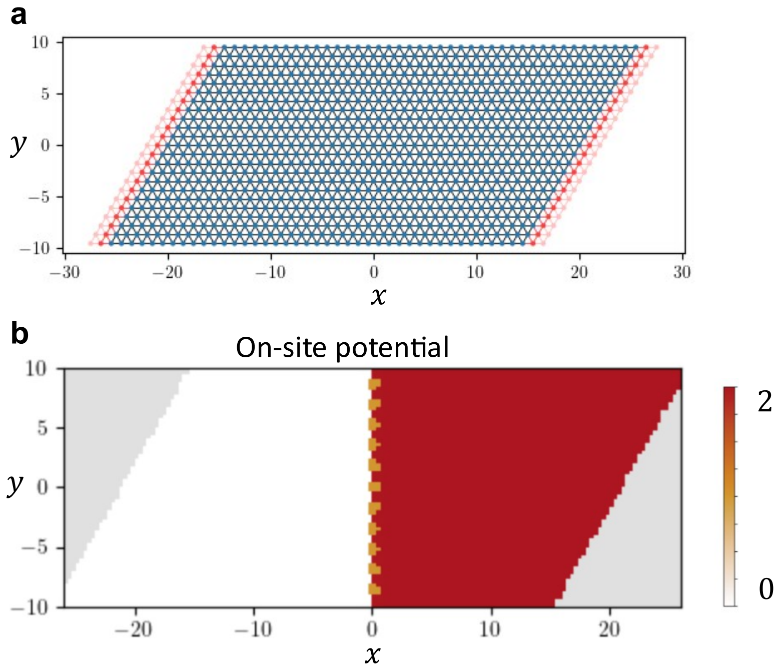


**Supplementary Figure 5. The nanoribbon p-n junction. a,** The lattice, consisting of a finite region (blue) and two leads (red). **b,** The on-site potential energy profile of an example p-n junction with step-function transition.

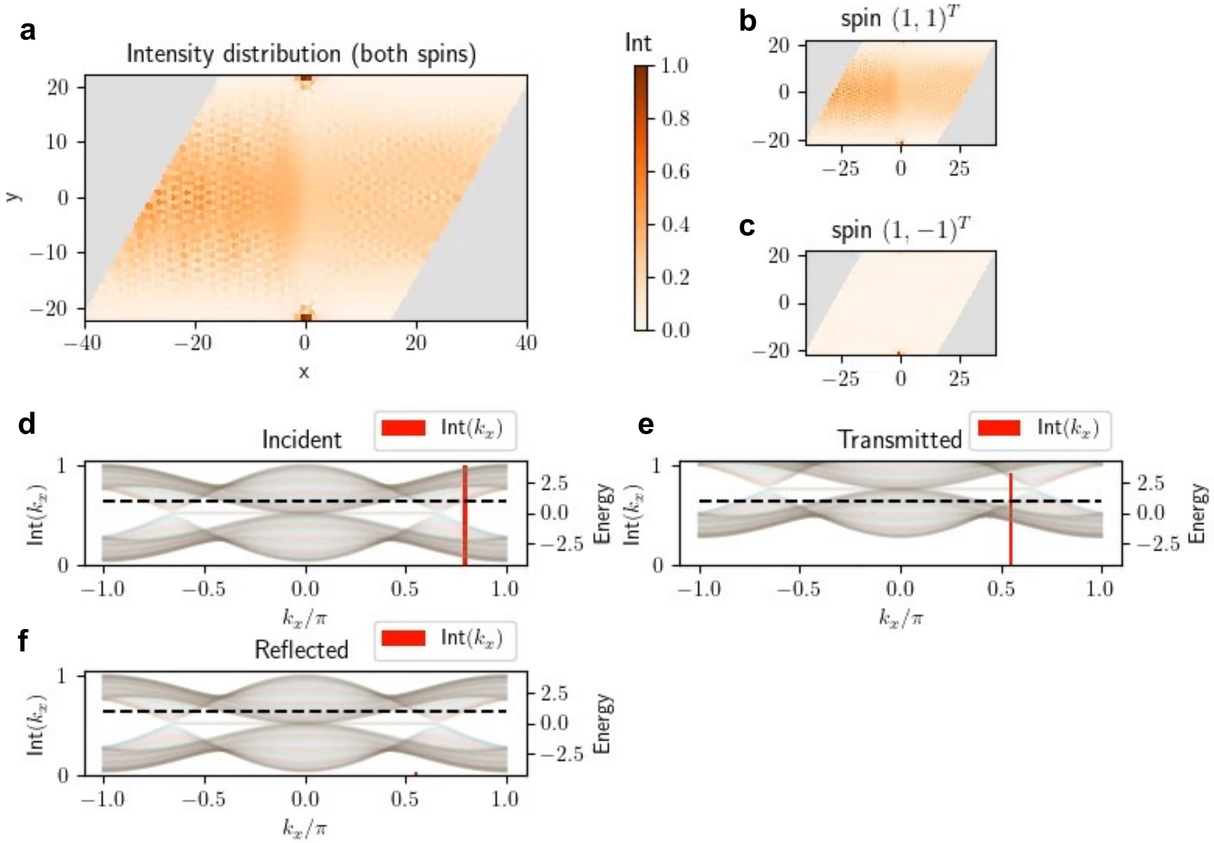


**Supplementary Figure 6. Scattering simulation in a nanoribbon p-n junction with width of 43 and incident wave being the fundamental mode in the Dirac valley, demonstrating the Klein tunneling with transmission coefficient of 96%. a–c,** The intensity distribution of both spins, spin $(1,1)^T$, and spin $(1,-1)^T$, respectively. **d–f,** The intensity distribution (red bars) of the incident, transmitted, and reflected waves, in the basis of port eigenmodes labeled by their $k_x$. Band structure calculated in the semi-infinite lead regions are overlayed in the background. The black dashed line indicates the energy of the incident wave.

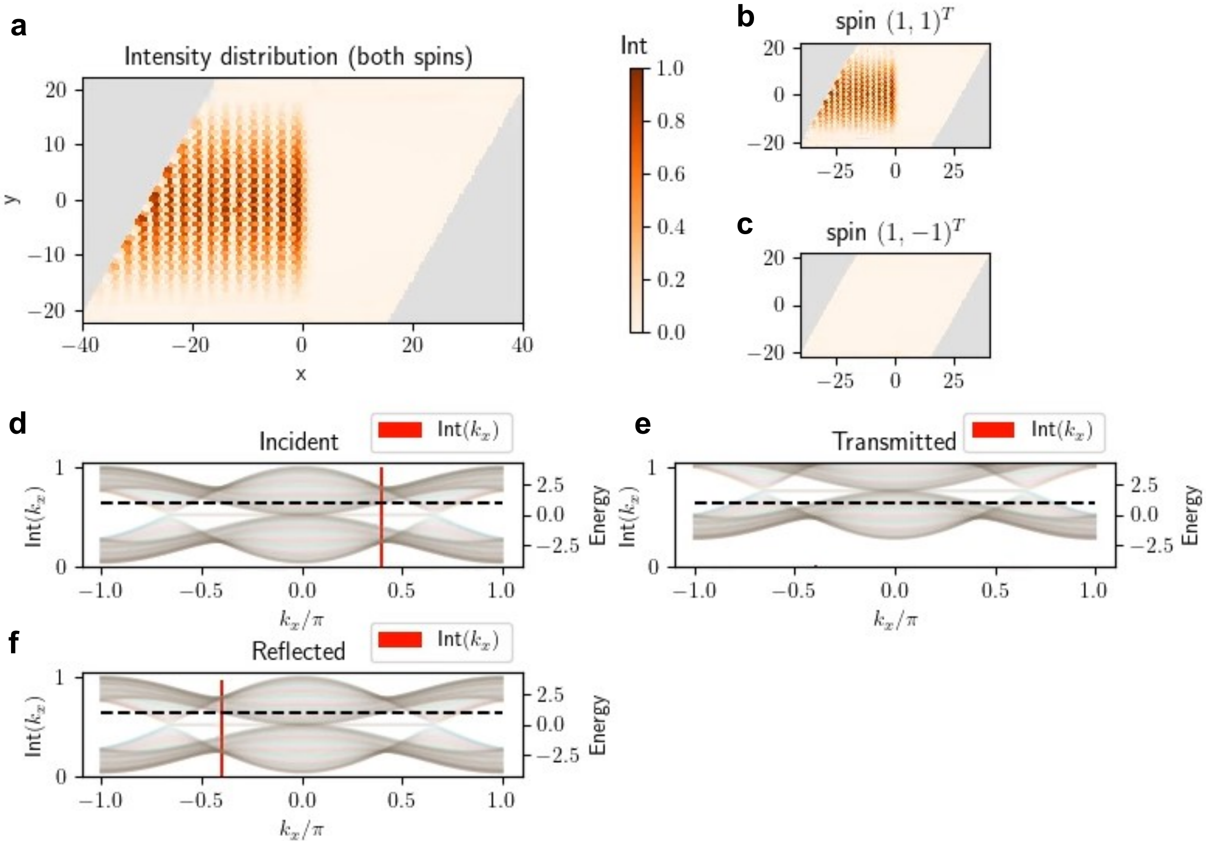


**Supplementary Figure 7. Scattering simulation in a nanoribbon p-n junction with width of 43 and incident wave being the fundamental mode in the quadratic valley, demonstrating the anti-Klein tunneling with reflection coefficient of 99%. a–c,** The intensity distribution of both spins, spin $(1,1)^T$, and spin $(1,-1)^T$, respectively. **d–f,** The intensity distribution (red bars) of the incident, transmitted, and reflected waves, in the basis of port eigenmodes labeled by their $k_x$. Band structure calculated in the semi-infinite lead regions are overlayed in the background. The black dashed line indicates the energy of the incident wave.

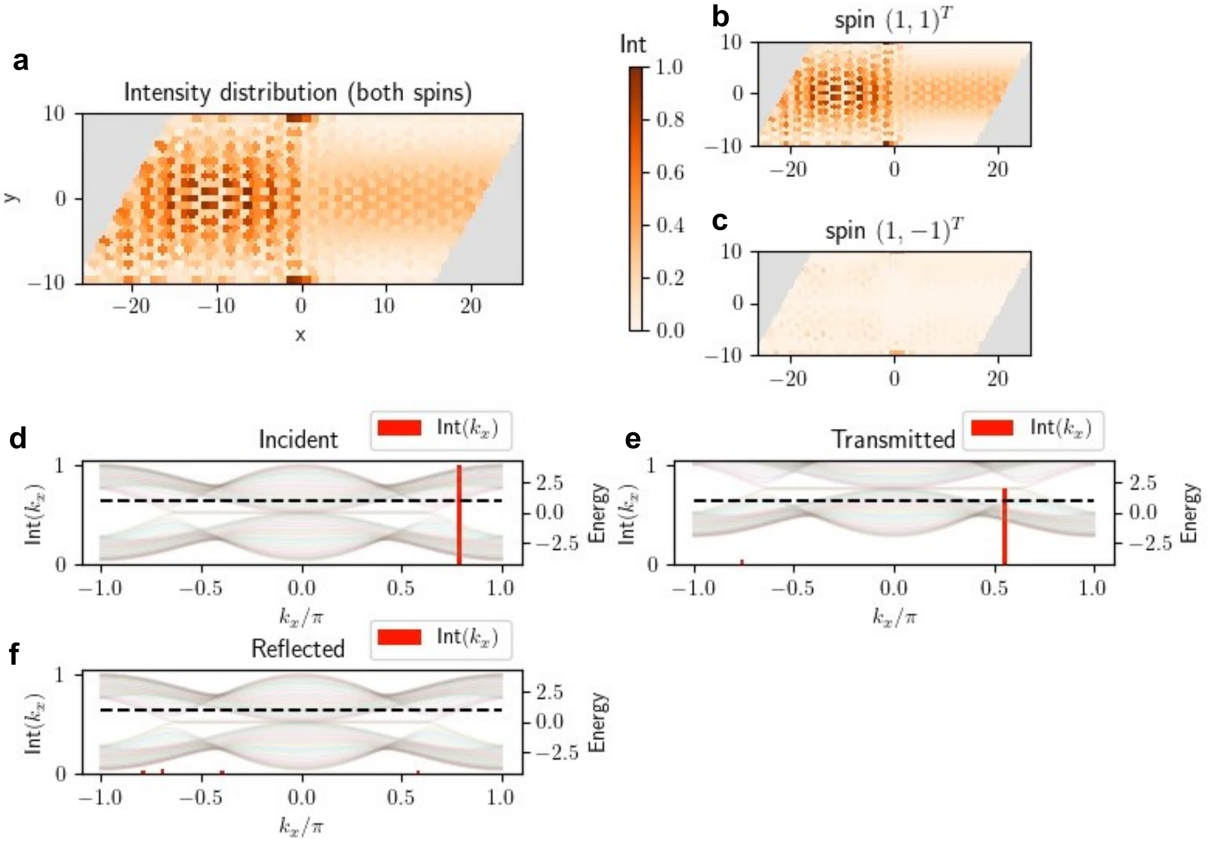


**Supplementary Figure 8. Klein-tunneling simulation in a nanoribbon p-n junction with width of 19 and step-function potential barrier.** The incident wave is the fundamental mode in the Dirac valley, demonstrating Klein tunneling with transmission coefficient of 88%. **a–c,** The intensity distribution of both spins, spin $(1,1)^T$, and spin $(1,-1)^T$, respectively. **d–f,** The intensity distribution (red bars) of the incident, transmitted, and reflected waves, in the basis of port eigenmodes labeled by their $k_x$. Band structure calculated in the semi-infinite lead regions are overlayed in the background. The black dashed line indicates the energy of the incident wave.

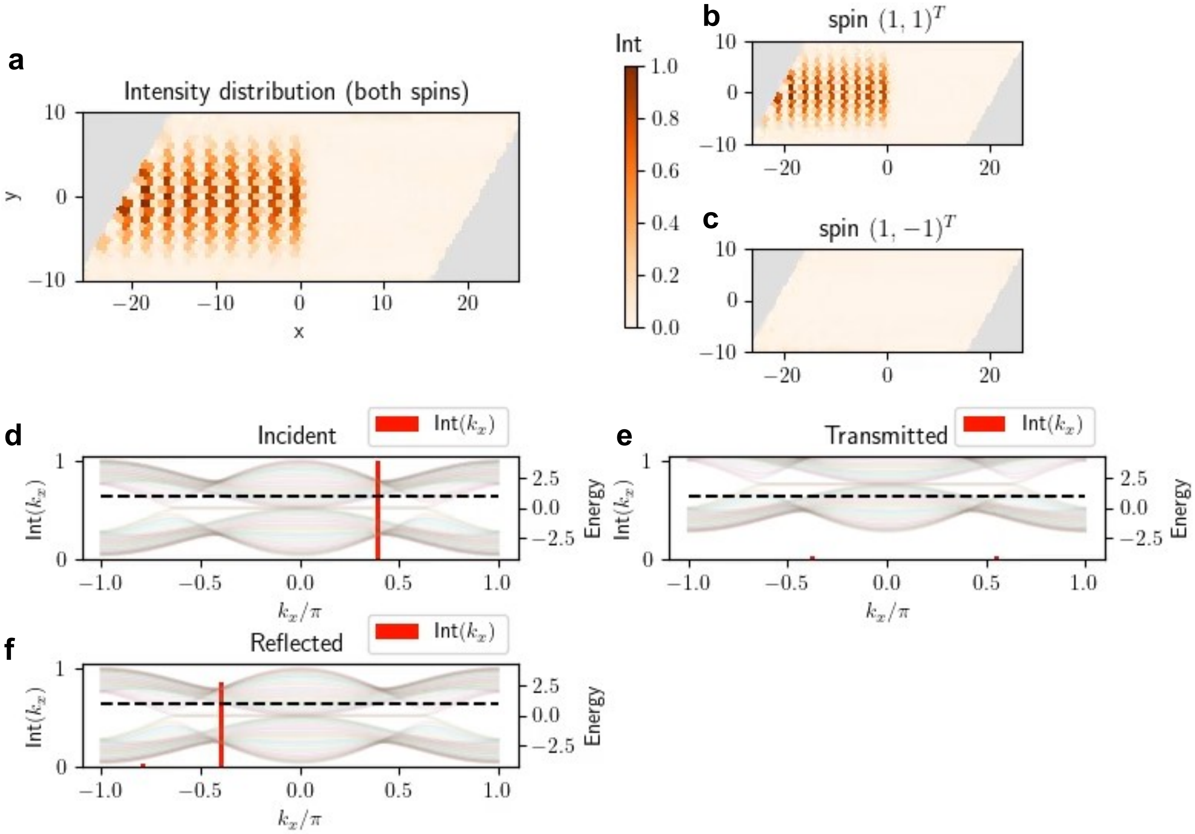


**Supplementary Figure 9. Anti-Klein tunneling simulation in a nanoribbon p-n junction with width of 19 and step-function potential barrier.** The incident wave is the fundamental mode in the quadratic valley, demonstrating anti-Klein tunneling with reflection coefficient of 93%. **a–c,** The intensity distribution of both spins, spin $(1,1)^T$, and spin $(1,-1)^T$, respectively. **d–f,** The intensity distribution (red bars) of the incident, transmitted, and reflected waves, in the basis of port eigenmodes labeled by their $k_x$. Band structure calculated in the semi-infinite lead regions are overlayed in the background. The black dashed line indicates the energy of the incident wave.

## 4. Edge-excited quadratic-node nanoribbon p-n junction

Since experimentally we excite the modes in the nanoribbon with edge excitation, here we show tight-binding-model calculation with edge excitations of uniform strength (instead of injecting eigenmodes as we did in Section 3). Here we show that with uniform excitation, we can excite both the quadratic and the Dirac valleys and therefore both the massive and massless components for a single chiral particle, which undergoes Klein and anti-Klein tunneling respectively.

Consider coupled-mode theory with excitations, the temporal evolution of the wavefunction can be written as

$$\frac{d}{dt}\begin{pmatrix}\psi_1\\ \psi_2\\ \vdots\\ \psi_N\end{pmatrix} = -\mathrm{i}\begin{pmatrix}\omega_1 & -\kappa_{12} & -\kappa_{13} & \cdots\\ -\kappa_{21} & \omega_2 & -\kappa_{23} & \ddots\\ -\kappa_{31} & -\kappa_{32} & \ddots & \ddots\\ \vdots & \ddots & \ddots & \omega_N\end{pmatrix}\begin{pmatrix}\psi_1\\ \psi_2\\ \vdots\\ \psi_N\end{pmatrix} + \begin{pmatrix}s_1\\ s_2\\ \vdots\\ s_N\end{pmatrix} = -iH\Psi + S \tag{8}$$

Assuming harmonic excitation with angular frequency of $\omega$ and wavefunction temporal dependence of $\frac{d}{dt}\Psi = (-i\omega)\Psi$, we have $(H - \omega I)\Psi = -iS$ and then $\Psi = -i(H - \omega I)^{-1}S$.

Again, we use software package Kwant to setup the finite lattice region and calculate the tight-binding Hamiltonian $H$, then we numerically solve the matrix inversion and steady-state wavefunction under excitation $S$. We set the spin of the excitation to $|+\rangle_x = (1,1)^T$, and the energy of excitation to 0.5. The intensity distribution of the wavefunction is shown in Supplementary Figure 10. The intensity distribution in reciprocal space is calculated using hexagonal fast Fourier transform [4]. Excitation of the $|-\rangle_x$ spin is much weaker compared with the$|+\rangle_x$ spin, and we observe simultaneous Klein and anti-Klein tunneling.

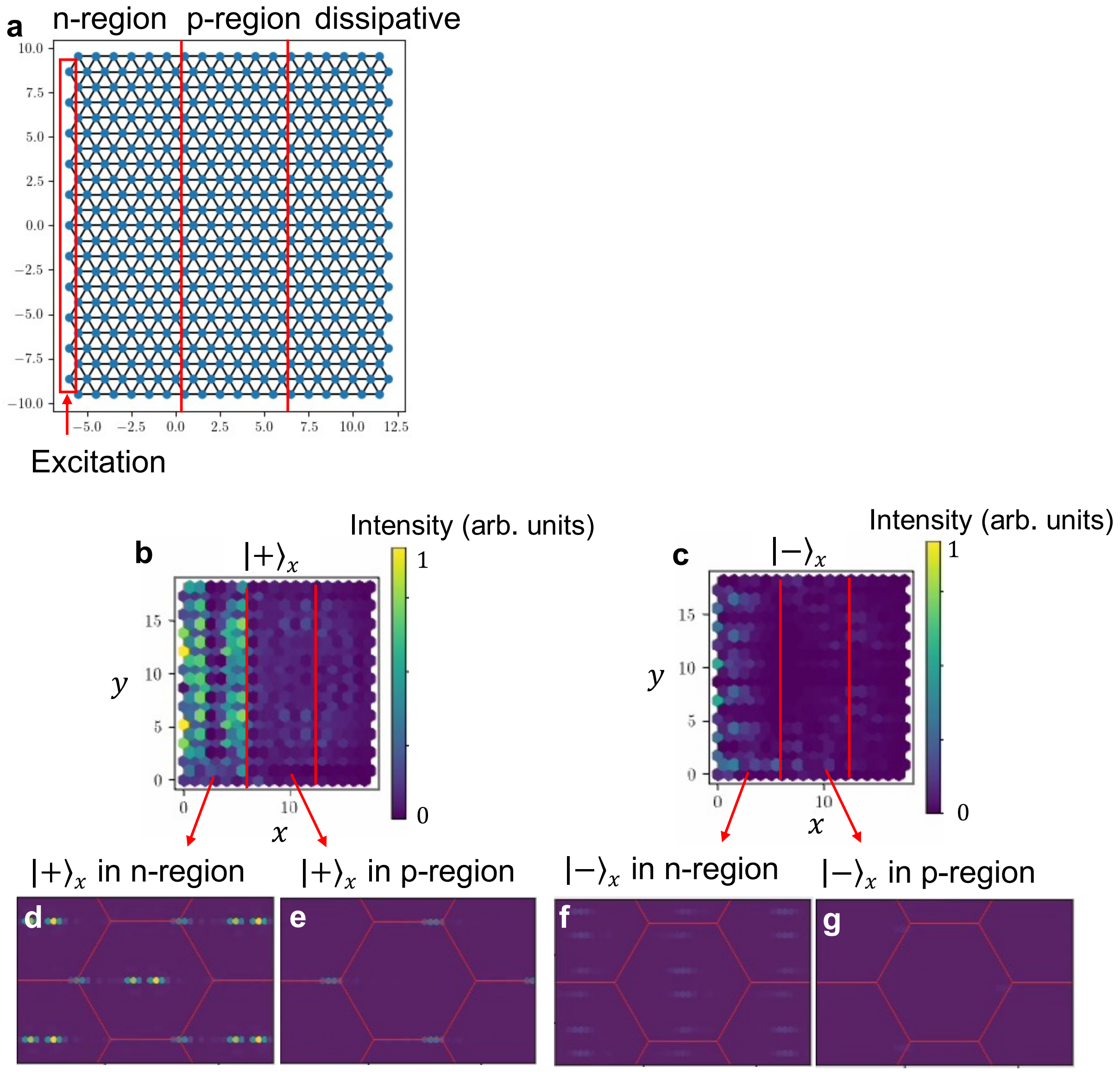


**Supplementary Figure 10. Simultaneous Klein an anti-Klein tunneling in an edge-excited nanoribbon p-n junction. a,** The p-n junction with edge excitation and a dissipative absorber region. **b & c,** Wavefunction intensity distribution in real space, for the $|\pm\rangle_x$ spins respectively, plotted with the same color scale for both spins. **d–g,** Wavefunction intensity distribution in the reciprocal space, plotted with the same color scale. Anti-Klein tunneling is observed in **a** near the Γ point, and Klein tunneling is observed in **d** and **e** near the K′ point.

## 5. Full wave finite-element method simulation of the photonic quadratic-node semimetal

We simulate the band structure and the transport properties with full wave finite-element method (FEM) using commercial software COMSOL. For the band structure, we simulate a single unit cell with periodic boundary conditions. For each k-vector that we sample along the edge of the irreducible Brillouin zone (BZ), we extract the eigenmode frequencies for the corresponding mode orders and plot them in Supplementary Figure 11a-c. For the case of weak coupling (with 600-nm gap between microrings, Supplementary Figures 11a and S11b), we observe particle-hole-like symmetry in the band structure. For the case of strong coupling (with 200-nm gap between microrings, Supplementary Figure 11c), the quadratic band touching and the Dirac band touching have an offset in energy. It can also be observed that the intensity distribution in the strong coupling case is disturbed by the coupling (Supplementary Figure 11e), which also indicate deviation from the ideal case of coupled-mode theory which requires the coupling to be weak enough to not change the resonant mode itself (satisfied for the weak coupling case as shown in Supplementary Figure 11d). However, experimentally the strong coupling is favorable to overcome the detuning from imperfect fabrication quality limited by our fabrication facility. Also, from the transport simulations (for example, comparing Supplementary Figure 10 and Supplementary Movie 8), we observe that the transport properties under study to be qualitatively independent of the band distortion induced by strong coupling.

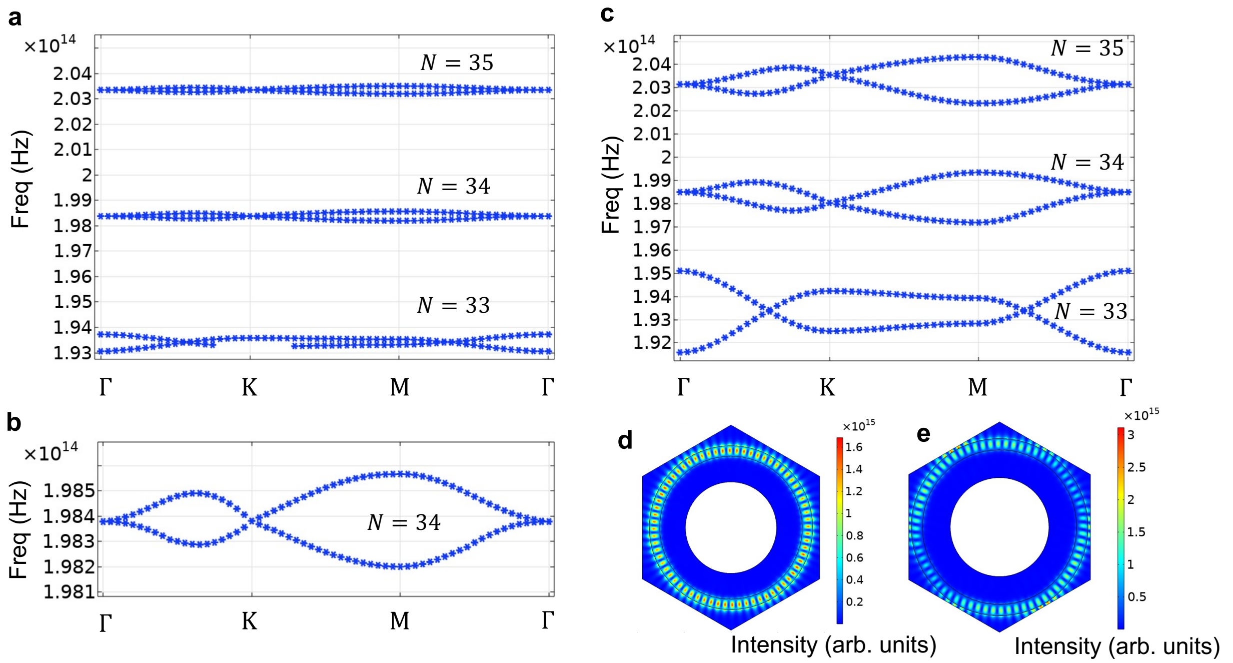


**Supplementary Figure 11. The band structure and the intensity distribution in the unit cell with 2D FEM simulation. a,** Band structure, in the case of weak coupling (600-nm gap between microring resonators). **b,** Zooming into the mode order 34 for the case of weak coupling, showing particle-hole-like symmetry. **c,** Band structure, in the case of strong coupling (200-nm gap between microring resonators). **d & e,** Intensity distribution in the unit cell at $M$ point for the lower branch of mode order 34, for weak and strong coupling, respectively.

For the transport properties, we model the coupled microring resonators with 2D effective index profile shown in Supplementary Figure 12. When modeling the p-n junction, the configuration is the same as in the experiment (shown in Fig. 4a), with 6 columns of n-type rings (radius of 450 nm in 2D simulation), followed by 6 columns of p-type rings (radius of 443 nm in 2D simulation), then terminated with dissipative

rings with increasing loss. When modeling the homogeneous quadratic-node semimetal, all the rings are set to have radius of 450 nm. Excitation is implemented with scattering boundary condition with $H_z$ incident field, where $z$ is the out-of-plane direction. We set incident field to be in-phase to excite the $(1,1)^T$ pseudospin. The $H_z$ field amplitude is then sampled in each ring to give us $H_z(m,n)$ where $(m,n)$ labels the location of the ring in the lattice. $H_z(m,n)$ can then be Fourier transformed into reciprocal space.

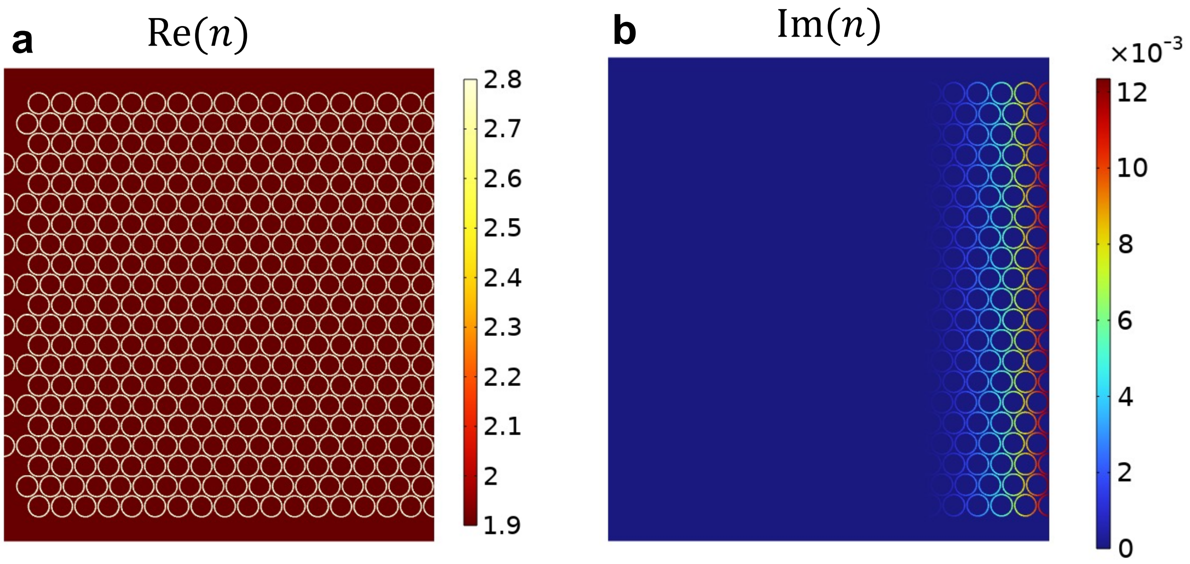


**Supplementary Figure 12. Refractive index profile in the FEM simulation. a,** Real part of the refractive index. **b,** Imaginary part of the refractive index.

**6. Electric field distribution emitted by an individual ring in the Fourier space**

The full field distribution in the photonic lattice or nanoribbon device can be approximated by the convolution of the field distribution of a single ring, $\mathbf{E}_s(\mathbf{r})$, with the discretely sampled field amplitude defined on the triangular lattice, $A(m,n)$

$$\begin{aligned}\mathbf{E}(\mathbf{r}) &= \mathbf{E}_s(\boldsymbol{r}) * \sum_{m,n} A(m,n)\delta(\mathbf{r} - m\mathbf{a}_1 - n\mathbf{a}_2) \\ &= \int \mathbf{E}_s(\mathbf{r}') \cdot \sum_{m,n} A(m,n)\delta(\mathbf{r} - m\mathbf{a}_1 - n\mathbf{a}_2 - \mathbf{r}')\, d\mathbf{r}' \\ &= \sum_{m,n} A(m,n)\mathbf{E}_s(\mathbf{r} - m\mathbf{a}_1 - n\mathbf{a}_2) \qquad (9)\end{aligned}$$

where $m, n$ labels the location of the lattice site relative to the coordinate origin in terms of lattice vectors $\mathbf{a}_{1,2}$. The Fourier transform (FT) of this field distribution can be written as

$$\mathbf{E}(\mathbf{r}) \overset{\text{FT}}{\rightarrow} \tilde{\mathbf{E}}_s(\mathbf{k}) \cdot \tilde{A}(\mathbf{k}) \qquad (10)$$

This tells us that the electric field in the far field is the product of the single ring far field $\tilde{\mathbf{E}}_s(\mathbf{k})$ and the array factor $\tilde{A}(\mathbf{k})$. Because in the device plane, $\mathbf{E}_s(\mathbf{r})$ is of smaller scale compared to $A(\mathbf{r}) = \sum_{m,n} A(m,n)\delta(\mathbf{r} - m\mathbf{a_1} - n\mathbf{a_2})$ defined on a large-scale array, the Fourier transform of them, $\tilde{\mathbf{E}}_s(\mathbf{k})$ and $\tilde{A}(\mathbf{k})$, control the a large-scale envelope and the fine scale detail, respectively, in the far field. Due to the discreteness of $A(\mathbf{r})$, $\tilde{A}(\mathbf{k})$ is repetitive in each BZ.

The single ring far field with angular gratings acting as scatters is shown in Supplementary Figure 13. For CW (CCW) traveling modes, the scattered field is in right- (left-) handed circular polarization[5]. This mapping between power flow direction and the polarization of emission can play an important role when the photonic lattice forms coupling with other structures, for example, another layer of metasurface, 2D photonic lattice, or 2D material, to form bi-layer structures. This mapping can be freely controlled by the angular locations of the scatters, which is an advantage of our system. When the number of scatters per resonator (i.e., grating order) does not equal to the mode order, this change of mapping would not affect the light flow (or effective Hamiltonian) within the lattice. However, if the grating order matches the mode order, then there exist off-diagonal terms in the on-site energy, which represents the cross-spin coupling (i.e., spin-flipping process) within a single ring.

In addition to the circular polarization, the far field of CW (CCW) traveling modes also carries orbital angular momentum of order $N - M - 1$, where $N, M$ are the mode order and the grating order [5]. For devices of mode order 34 and grating order 33 (corresponding to Fig. 3d and 3f, and Fig. 4), the single ring far field carries no orbital angular momentum, and the field and polarization distribution is plotted in Supplementary Figure 13a and S13b for the $(1, \pm 1)^T$ pseudospins respectively. The two pseudospins emit in orthogonal linear polarizations, since the equal superposition of two circular polarizations yields linear polarization. In our grating design, we align the gratings so that there is always a scatter at $\Phi = 0$ (Supplementary Figure 13e). This way, based on our definition of the phase origin (see Section 1), $(1, \pm 1)^T$ always results in vertical and horizontal polarizations, respectively. If we were to rotate the grating location along the ring by $\Delta\Phi$, the polarization orientation emitted by the two pseudospins would also rotate by $N\Delta\Phi$, where $N$ is the mode order.

For mode order 35 and grating order 33 (corresponding to Fig. 3c and 3e), the single ring far field carries orbital angular momentum of 1. In this case, the far field polarization distribution is no longer spatially uniform. Instead, the two pseudospins emits azimuthal and radial polarizations, respectively [5], shown in Supplementary Figure 13c and S13d. However, for a specific location in the BZ, we can still

approximate the local polarization to be linear. More specifically, it can be observed that along $k_y = 0$, the polarization emitted by the two pseudospins are identical to the case of mode order 34. Since all the cones we characterized in Fig. 2 and Supplementary Movies 1–3 are along $k_y = 0$, the Bloch sphere in Fig. 2c applies to all of them.

It can be observed that the mode order 34 has stronger emission at the center of the first BZ, while the mode order 35 has stronger mission along the edge of the first BZ, therefore we characterize the quadratic band touching using mode order 34 and characterize the Dirac band touching using mode order 35 for better signal-noise ratio.

The single ring far field also impacts our characterization of transport in nanoribbons. We perform the Fourier space imaging of the wavefunction in the hexagonal BZ that is next to (above) the first BZ (because experimentally we observe strong stray light in the first BZ, possibly from light leaking out of the multi-mode interferometers and subsequently scattered by the rings). With the cross-spin polarization filtering, we measure only the vertically polarization emission. In this configuration, for mode order 35, any signal around the $k_x = 0$ is attenuated since it is horizontally polarized (Supplementary Figure 13c), which is consistent to what we experimentally observed. Also, in the BZ directly above the first one, from Supplementary Figure 13d, it is expected that the measured signals for $N = 35$ are stronger in the lower half of the BZ, which is indeed what we observe in Fig. 3c. However, we also note that in the experiments, the scattered field intensity at each scatter may not be perfectly identical due to fabrication-imperfection-induced scatter size difference and nonuniform intensity distribution in strongly coupled ring lattices. As a result, the destructive interference may not be perfect, meaning we may still see nonzero field at the location of dark lines.

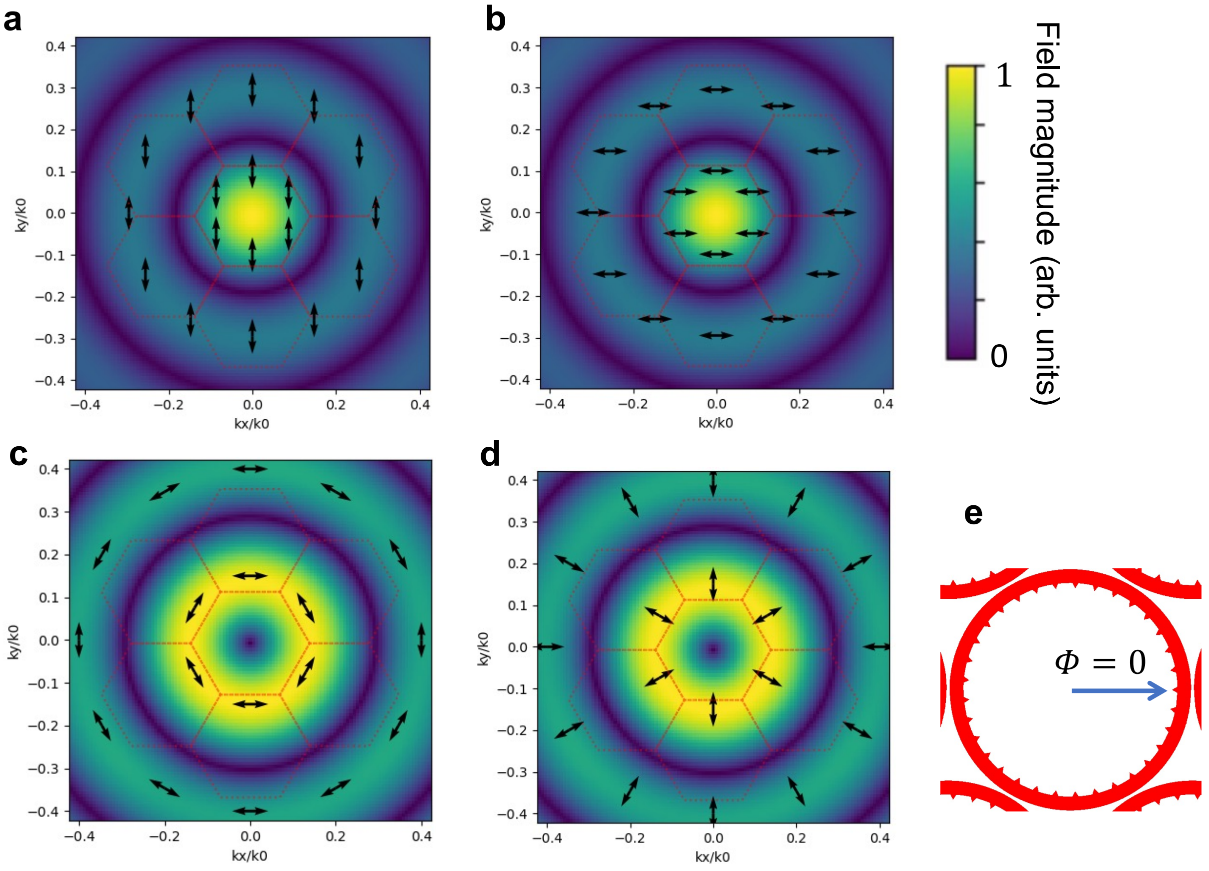

**Supplementary Figure 13. Electric field magnitude (pseudo color) and polarization distribution (black arrows) of the single ring far field, overlayed with the Brillouin zones of the triangular lattice (red dotted lines). a,** $(1,1)^T$ pseudospin with mode order 34 and grating order 33. **b,** $(1,-1)^T$ pseudospin with mode order 34 and grating order 33. **c,** $(1,1)^T$ pseudospin with mode order 35 and grating order 33. **d,** $(1,-1)^T$ pseudospin with mode order 35 and grating order 33. **e,** Grating orientation used in the calculation, with the first scatter aligned at $\Phi = 0$. For **a–d**, the x and y axes are the wavevectors in $x$ and $y$ directions normalized to the free-space wavevector $k_0$.

## 7. Stray light in cross-polarization reflection characterization due to imperfect cross-polarization filtering

Majority of the noise we observe in Fig. 2 and Supplementary Movies 1–2 is from the direct reflection and diffraction of the incident beam that does not excite the confined mode in the lattice. When we use free-space plane waves to excite the resonant modes in the ring array, the excitation efficiency is low as a result of poor modal overlap, since the ring array has large void areas inside and between the rings. The majority of the incident beam does not excite the modes inside the photonic lattice and the direct reflection and diffraction of this incident beam becomes stray light. We filter out most of this stray light with cross-polarization filtering. However, this polarization filtering is perfect only at normal incidence. Hence the polarization filtering works best at the center of the first BZ, and consequently the Dirac cones at the corner of the BZ have lower signal-to-noise ratio due to imperfect filtering of the stray light.

We can also see that the emission emerging from other BZs contains more stray light than the center BZ because of the same reason (i.e., the cross-polarization filtering that is responsible for eliminating the stray light performs best only at normal angle). The signals from Bloch modes in the photonic lattice are buried underneath this stray light, making them difficult to observe. Diffraction can be understood as the microring lattice providing in-plane momentum $\mathbf{G} = m_1\mathbf{b_1} + m_2\mathbf{b_2}$ to diffract the incident beam to other BZs in the far field image. Here $\mathbf{b_{1,2}}$ are the primitive wavevectors of the reciprocal lattice. At BZs other than the center one, the diffraction angle slightly rotates the polarization and adds nonzero component to the orthogonal polarization direction, which will not be filtered out by the analyzer and consequently appears in the Fourier-space images. However, this polarization rotation does not happen when the polarization orientation is orthogonal to or aligned with the in-plane momentum **G**. Hence, we do observe the appropriate polarization patterns at other BZs when the polarizer and analyzer orientations happen to be aligned with the corresponding **G**. This can be observed, for example, in Supplementary Movie 1 when polarizer is at 120° and 240°.

## 8. Transport characterization of the homogeneous quadratic-node semimetal nanoribbon

Here in Supplementary Figure 14 we provide the Fourier plane images and full wave simulation for the data points shown in Figure 3 (except the ones with experimental and simulated Fourier space images already shown in Fig. 3). In addition, we show the process to extract the $k_x$ locations marked by the green stars in Figs. 3, 4 and Supplementary Figs. 14 and 15.

For Figs. 3e-f, the positions of the green starts are defined as the positions of the intensity peak(s) in the Fourier-space images along the $k_y = 0$ line cut (see Supplementary Figure 14e). The protocol is to extract the highest intensity peak first. If there is a second peak visible from the Fourier-space image, then we also plot the second peak. Because of the limited signal-to-noise ratio (here the noise also includes stray light scattered from the input grating coupler and the multi-mode interferometers), it is easier to distinguish the signal from the stray light in the 2D Fourier-space images than in the 1D line cuts. Identification of the "second highest peak" may not be very obvious due to limited signal-to-noise ratio. However, for the two wavelengths shown in Fig. 3d, the second highest peaks can be clearly identified, especially when

comparing with the simulated Fourier-space images. Hence, we choose to plot these second-highest peaks as well.

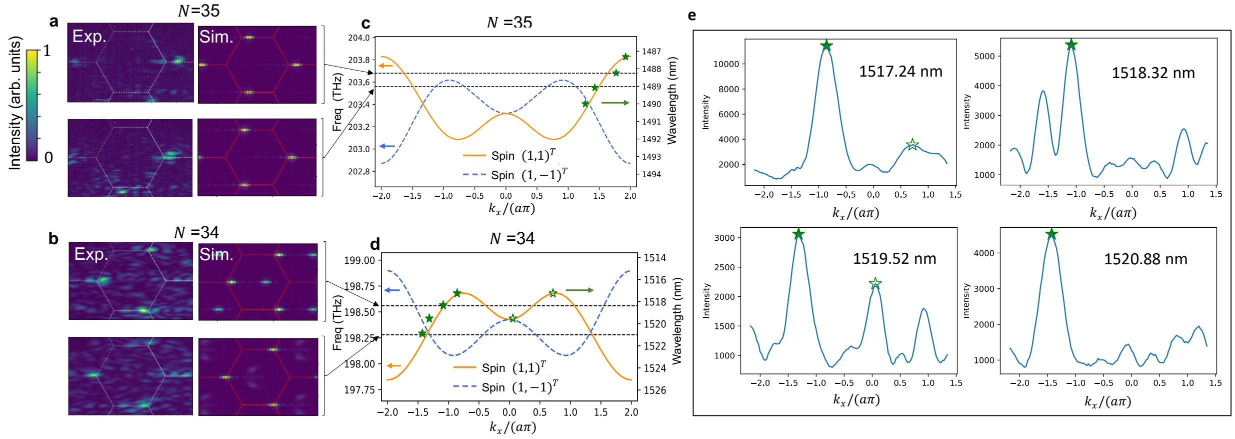


**Supplementary Figure 14. Experimental Fourier plane imaging and full wave simulation of the wavefunction intensity distribution in an edge-excited homogeneous quadratic-node semimetal nanoribbon. a,** Experimental and simulated Fourier domain intensity distributions for mode order 35, at wavelength of 1488.24 nm and 1489.08 nm (experimental images) and frequency of 203.68 THz and 203.56 THz (simulation). **b,** Experimental and simulated Fourier domain intensity distributions for mode order 34, at wavelength of 1518.32 nm and 1520.88 nm (experimental images) and 198.56 THz and 198.28 THz (simulation). **c & d,** $k_x$ extracted from the experimental images at the corresponding wavelengths (green stars, extracted from both the images shown here and the images in Fig. 3), overlayed on the simulated band structure, with the left y-axis to be the simulation frequency and the right $y$-axis to be the wavelength in experiments. The two-axes are not exactly aligned to each other, but the quantitative agreement between experimentally measured and the simulated band structure can be observed. **e**, Cross sections of the Fourier-space images along $k_y = 0$, for $N = 34$. The solid (hollow) green stars show locations of the highest (second-highest) intensity peaks, which are also plotted in **d** and in Fig. 3.

## 9. Transport characterization of the quadratic-node semimetal nanoribbon p-n junction

Here in Supplementary Figure 15 we provide the Fourier plane images and full wave simulation for several data points in addition to the ones shown in Fig. 4.

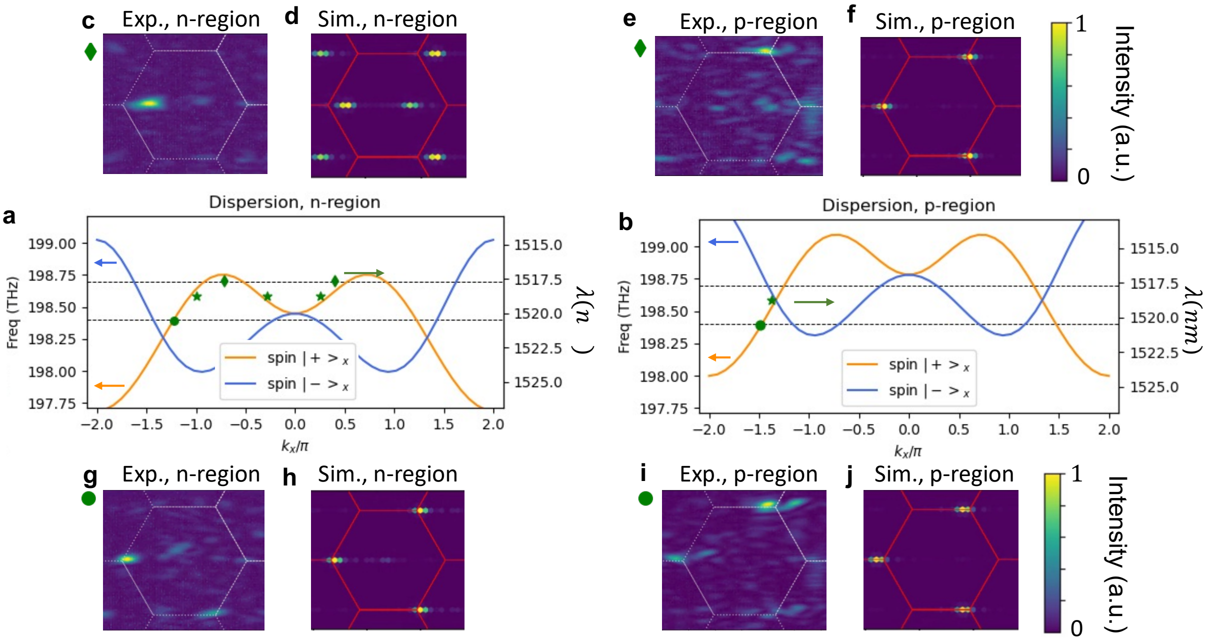


**Supplementary Figure 15. Experimental Fourier plane imaging and full wave simulation of the wavefunction intensity distribution in edge-excited quadratic-node semimetal nanoribbon p-n junction. a & b,** Simulated band structure in n- and p-regions, respectively (left $y$-axes) with experimentally measured data points (green symbols, incident wavelengths on right y-axes). **c–f,** Experimental and simulated Fourier domain intensity distributions at wavelength of 1517.60 nm (experimental images) and frequency of 198.7 THz (simulation), in the n- and p-regions, respectively. **g–j,** Experimental and simulated Fourier domain intensity distributions at wavelength of 1520.52 nm (experimental images) and frequency of 198.4 THz (simulation), in the n- and p-regions, respectively. The energy levels in the experiment and the simulation are not exactly aligned to each other, but the quantitative agreement between experimentally measured and the simulated band structure can be observed.

## 10. The cross-spin and same-spin coupling in coupled microrings

The cross-spin coupling differentiates the quadratic-node semimetal from many other lattice systems. The cross-spin coupling is a result of momentum (or equivalently, wavevector $\beta$) or power flow conservation during the coupling process between adjacent rings. Intuitively, this is depicted in Fig. 1b by the green and red arrows indicating power flow directions. In the following, we estimate the ratio between cross-spin coupling and same-spin coupling analytically to gain insight into the physics that underpins it and show full-wave simulation that agrees with the analytical approximation.

We calculate the coupling coefficient by evaluating the mode overlap between adjacent rings[6]. By abstracting the coupling region as two parallel waveguides (see Supplementary Figure 16a), we can estimate the mode overlap (for example, the overlap between $H_z$ components in a 2D model). Because our goal is to derive the ratio between cross-spin coupling and same-spin coupling, we drop some of the normalization factors and write the mode overlap as

$$\kappa \sim \frac{1}{2\pi R}\int_0^L \left(e^{i\beta_1 l}e^{-\gamma d}\right)^* e^{i\beta_2 l}dl = -\frac{ie^{-\gamma d}}{2\pi R}\frac{e^{i(\beta_2-\beta_1)L}-1}{\beta_2-\beta_1} \quad (11)$$

where $\beta_{1,2}$ are the wavevectors in the left and right waveguides, respectively, and they can be of the same sign (for cross-spin coupling) or opposite signs (for same-spin coupling). $L$ is the length of the coupling region, $d$ is the gap between waveguides, $\gamma$ is the decay rate of the field amplitude in the cladding, and $l$ is the local coordinate along the waveguide. For cross-spin coupling, we have $\beta_1 = \beta_2$ and $|\kappa| = \frac{e^{-\gamma d}L}{2\pi R}$. For same-spin coupling, we have $\beta_1 = -\beta_2 = \beta$ and $|\kappa| = e^{-\gamma d} \frac{|e^{2i\beta L}-1|}{4\pi R\beta}$. The amplitude ratio between same-spin and cross-spin coupling is therefore

$$\frac{|\kappa_{\text{same-spin}}|}{|\kappa_{\text{cross-spin}}|} = \frac{|e^{i2\beta L} - 1|}{2\beta L} \tag{12}$$

This model assumes adiabatic transition into and out of the coupling region and does not capture the non-adiabatic nature of transition when the radius of the microring is very small. However, it intuitively shows that the momentum conservation in cross-spin coupling manifest as the linear increase of coupling strength with longer coupling length $L$. On the other hand, the same-spin coupling has momentum mismatch of $2\beta$ and the coupling strength oscillates around a constant. Therefore, when the WGM mode order is large, the long coupling region ensures that the cross-spin coupling dominates. We perform full-wave simulation using COMSOL and show the ratio between same-spin and cross-spin coupling in Supplementary Figure 16. The decrease of same-spin coupling relative to cross-spin coupling is approximately radius$^{-2}$ in terms of power ratio. Since the effective coupling length scales approximately linearly with the radius of the microrings (Supplementary Figure 16c), this simulation result agrees with the analytical estimation.

In summary, the ratio between same-pin and cross-spin coupling decreases approximately quadratically with the radius of the microring, and in our system, with a radius of 3.5 µm, this ratio is sufficiently small (about 0.3% in terms of power).

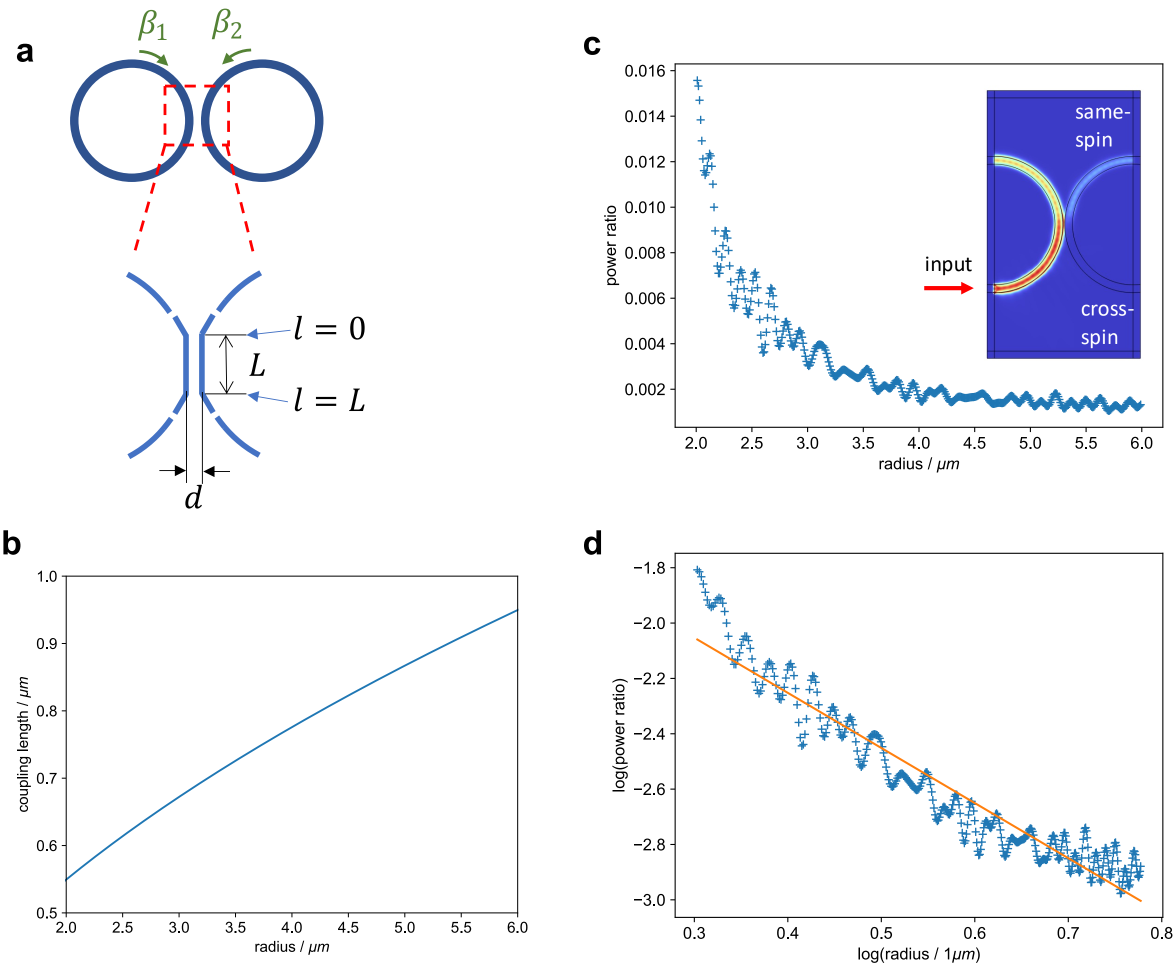


**Supplementary Figure 16. The relative strengths of cross-spin and same-spin coupling. a,** Schematic of the coupling region and the approximated geometry for analytical estimation. **b,** the coupling length $L$ versus the radius of the microrings. Here $L$ is defined as the length of the arc within the region where the gap between waveguides is less than 250 nm (arbitrarily chosen for estimation only). **c,** The power ratio between the same-spin and cross-spin coupling processes, simulated using COMSOL. The simulation setup is shown as inset, with an input port and two monitoring ports corresponding to same-spin and cross-spin processes, respectively. The whole structure is surrounded by perfectly-matched layers (PML). **d,** The log-log plot of power ratio versus ring radius (with radius from 2 μm to 6 μm). The orange line is the least-square linear fit showing a slope of −1.99.

## 11. Comparison between the quadratic-node semimetal and other example systems with linear or high-order nodes

There are various linear and high-order nodal points previously studied in condensed-matter materials as well as photonic and acoustic metamaterials. Some of them share similar local features compared to our quadratic-node semimetal, including quadratic dispersion and topological charge of two. We have summarized their differences in Supplementary Table 1. The quadratic-node semimetal reported here is the first 2D system featuring coexisting charge-2 and charge-1 nodes, distinct from any previously reported systems. The coexistence of quadratic and Dirac nodes, rooted in the cross-spin coupling and the commutation relations between symmetry operators, manifests profoundly the unique transport that we report here. For example, the coexisting massive and massless components in a chiral quasiparticle

(depicted in Fig. 3), the simultaneous Klein and anti-Klein tunneling (shown in Fig. 4), and the unique inter-valley dynamics (presented in Supplementary Information Section 2 and Supplementary Movies 4 and 5) are all tied to this unique nodal configuration.

In addition to this critical difference in nodal configuration, the quadratic-node semimetal represents the first 2D example with a quadratic node obeying the Nielsen–Ninomiya theorem. Specifically, our clockwise (CW) and counterclockwise (CCW) modes in the experimental setup exhibit only two bands. Due to space-time inversion symmetry, only in a *two-band* model the fragile topology[7] can arise and quantize charges of nodes across the entire BZ. Consequently, in our system, the total node charges are neutralized, as dictated by the Nielsen–Ninomiya theorem. This fundamental feature, associated with a quadratic node, is reported for the first time. In comparison, the photonic crystal systems with charge-2 quadratic degeneracies, for example, theoretically described in ref.[8], also preserve space-time inversion symmetry. However, despite the local node being described by an effective two-band model with protection from fragile topology, this two-band model cannot be extended throughout the entire BZ, rendering the Nielsen–Ninomiya theorem inapplicable. Also, the experimentally observed charge-2 band touching in photonic crystals, as exemplified in ref.[9,10] , can open up a gap when the nearby third band approach the degeneracy, demonstrating the fragility of the charge-2 node's topology[7].

To sum up, the quadratic-node semimetal is described by a two-band tight-binding model, much like the model of graphene except for the different nodal configuration, which is governed by the Nielsen–Ninomiya theorem. The theorem implies that the quadratic node is accompanied by two Dirac nodes. In contrast, previously reported photonic crystal systems are described by the limited low-energy theory valid only locally near the degeneracy points, making their physics distinct from ours. These distinctions, including system symmetries, the applicability of a two-band model, the validity of the Nielsen–Ninomiya (no-go) theorem, the existence and stability of the charge-2 and charge-1 nodes, are all summarized in Supplementary Table 1.

| System | $C_2T$ | Chiral | 2-band | No-go theorem | Charge-2 node | Charge-1 node |
|---|---|---|---|---|---|---|
| (2D) Quadratic-node semimetal | Yes | Yes for weak coupling | Yes | Yes | Stable, fragile | Stable |
| (2D) Graphene | Yes | Yes | Yes | Yes | Absence | Stable |
| (2D) Gated bilayer graphene[11] | No | No | No | No | Unstable | Absence |
| (2D) PhC pillar array[8] | Yes | No | Locally | No | Stable, fragile | Absence |
| (2D) PhC hole array[9,10] | Yes | No | Locally | No | Stable, fragile | Absence |
| (3D) Weyl semimetal[12] | No | No | No | Yes | Absence | Stable |
| (3D) Weyl semimetal[13] | No | No | No | Yes | Stable | Stable |

**Supplementary Table 1. Comparison between the quadratic-node semimetal and other example systems with linear or high-order nodes.** The columns summarize whether the system possess space-time inversion ($C_2T$) symmetry, chiral symmetry, can be described by a 2-band model, obeys the Nielsen–Ninomiya (no-go) theorem, and the existence and stability of the charge-2 and charge-1 nodes.

**12. Supplementary figures of the fabricated devices and the optical characterization set-up**

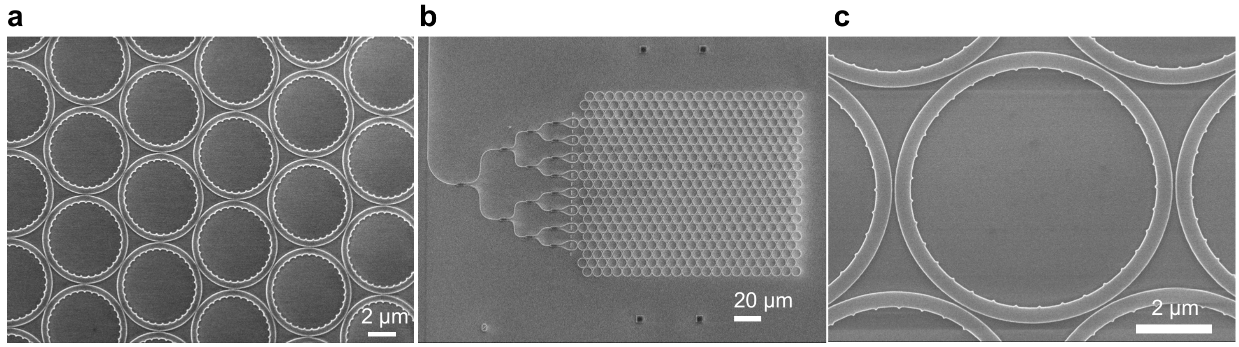


**Supplementary Figure 17. Scanning electron microscopy (SEM) images of the photonic quadratic-node semimetal. a,** Homogeneous quadratic-node semimetal with nominal scatter size of 180 nm, for cross-polarization reflection characterization. **b & c,** Quadratic-node nanoribbon p-n junction with edge excitation, the whole nanoribbon with input waveguides, and the zoomed-in view of a single ring (nominal scatter size of 80 nm), respectively.

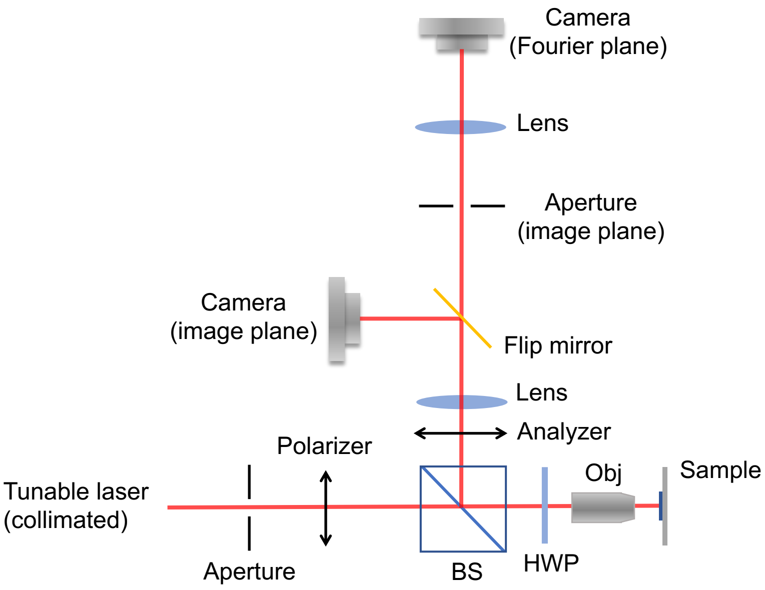


**Supplementary Figure 18. Optical set-up to characterize the photonic quadratic-node semimetal.** BS: beam splitter; HWP: half-wave plate; Obj: objective lens.

**Supplementary References:**

1. Wiersig, J. Structure of whispering-gallery modes in optical microdisks perturbed by nanoparticles. *Phys. Rev. A* **84**, 063828 (2011).

2. Johansson, J. R., Nation, P. D. & Nori, F. QuTiP 2: A Python framework for the dynamics of open quantum systems. *Comput. Phys. Commun.* **184**, 1234–1240 (2013).

3. Groth, C. W., Wimmer, M., Akhmerov, A. R. & Waintal, X. Kwant: a software package for quantum transport. *New J. Phys.* **16**, 063065 (2014).

4. Birdsong, J. B. & Rummelt, N. I. The hexagonal fast fourier transform. in *2016 IEEE International Conference on Image Processing (ICIP)* 1809–1812 (2016).

5. Shao, Z., Zhu, J., Chen, Y., Zhang, Y. & Yu, S. Spin-orbit interaction of light induced by transverse spin angular momentum engineering. *Nat. Commun.* **9**, 926 (2018).

6. Hong, J.-S. Couplings of asynchronously tuned coupled microwave resonators. *IEE Proc. - Microw. Antennas Propag.* **147**, 354 (2000).

7. Bzdušek, T. & Sigrist, M. Robust doubly charged nodal lines and nodal surfaces in centrosymmetric systems. *Phys. Rev. B* **96**, 155105 (2017).

8. Chong, Y. D., Wen, X.-G. & Soljačić, M. Effective theory of quadratic degeneracies. *Phys. Rev. B* **77**, 235125 (2008).

9. Zhang, Y. *et al.* Observation of Polarization Vortices in Momentum Space. *Phys. Rev. Lett.* **120**, 186103 (2018).

10. Chen, A. *et al.* Observing vortex polarization singularities at optical band degeneracies. *Phys. Rev. B* **99**, 180101 (2019).

11. Oostinga, J. B., Heersche, H. B., Liu, X., Morpurgo, A. F. & Vandersypen, L. M. K. Gate-induced insulating state in bilayer graphene devices. *Nat. Mater.* **7**, 151–157 (2008).

12. Xu, S.-Y. *et al.* Discovery of a Weyl fermion semimetal and topological Fermi arcs. *Science* **349**, 613–617 (2015).

13. Huang, S.-M. *et al.* New type of Weyl semimetal with quadratic double Weyl fermions. *Proc. Natl. Acad. Sci.* **113**, 1180–1185 (2016).
