# Supplementary material for: Topological quadratic-node semimetal in a photonic microring lattice
Source: Nat Commun. 2023 Jun 2;14:3206. doi: 10.1038/s41467-023-38861-3 (PMC10238381; doi:10.1038/s41467-023-38861-3)
Supplement: Supplementary file 3 — Description of Additional Supplementary Files [file 41467_2023_38861_MOESM3_ESM.pdf]

**Title: Supplementary Movie 1.**
**Description: Cross-polarization reflection near the quadratic band touching point with continuous rotation of polarizer and analyzer.** The pseudo color image represents reflected intensity image in the Fourier domain after cross-polarization filtering, with the dotted white line marking the first Brillouin zone and the double-headed arrows showing the orientations of polarizer and analyzer. The two dark lines near the Γ point in the reciprocal space mark the eigenstates with (pseudo) spin identical or orthogonal to the incident spin defined by the polarizer. With simultaneous rotation of the polarizer and analyzer orientations (implemented with a rotating half-wave plate), the rotation of these two dark lines marks the rotation of (pseudo) spin orientation in the reciprocal space and demonstrates the winding number of 2 (i.e., $2\pi$ Berry phase) around the quadratic band touching.

**Title: Supplementary Movie 2.**
**Description: Cross-polarization reflection near the Dirac band touching point with continuous rotation of polarizer and analyzer.** The pseudo color image represents reflected intensity image in the Fourier domain after cross-polarization filtering, with the dotted white lines marking the Brillouin zones and the double-headed arrows showing the orientations of polarizer and analyzer. The movie is centered at a K point. The single dark line near the K point in the reciprocal space mark the eigenstates with (pseudo) spin identical or orthogonal to the incident spin defined by the polarizer. With simultaneous rotation of the polarizer and analyzer orientations (implemented with a rotating half-wave plate), the rotation of this single dark line marks the rotation of (pseudo) spin orientation in the reciprocal space and demonstrates the winding number of 1 (i.e., $\pi$ Berry phase) around the Dirac band touching.

**Title: Supplementary Movie 3.**
**Description: Cross-polarization reflection near the quadratic band touching point with continuous scanning of incidence wavelength.** The pseudo color image represents reflected intensity image in the Fourier domain after cross-polarization filtering, with the dotted white line marking the first Brillouin zone and the double-headed arrows showing the orientations of polarizer and analyzer. Band touching can be observed around 1532 nm, with no energy gap (down to ~ 100 GHz resolution, limited by the ~1500 quality factor of rings with large scatters), consistent with point-touching nature of the semimetal band structure and our theory of a robust quadratic nodal point.

**Title: Supplementary Movie 4.**
**Description: Evolution of wavefunction in a p-n junction box, with initial pulse in the quadratic valley.** The p-n junction box (finite in $x$, infinite in $y$ direction) is terminated with open boundary arm-chair terminations, illustrated in Supplementary Figure 2. In the movie, top panel shows the intensity distribution in real space, and the two lower panels show the momentum-space intensity distribution in the n- and p-regions, respectively, overlaying on top of the band structure (right $y$-axis in each panel). The pulse first undergoes anti-Klein tunneling (perfect reflection) at the junction interface. Upon reflection at the open boundary, the pulse undergoes inter-valley scattering and becomes hybrid, and after which the component in Dirac valley and the component in quadratic valley show Klein and anti-Klein tunneling, respectively.

**Title: Supplementary Movie 5.**
**Description: Evolution of wavefunction in a p-n junction box, with initial pulse in the Dirac valley.** The p-n junction box (finite in $x$, infinite in $y$ direction) is terminated with open boundary arm-chair terminations, illustrated in Supplementary Figure 2. In the movie, top panel shows the intensity distribution in real space, and the two lower panels show the momentum-space intensity distribution in the n- and p-regions, respectively, overlaying on top of the band structure (right $y$-axis in each panel). The pulse first undergoes Klein tunneling (perfect transmission) at the junction interface. Upon reflection at the open boundary in the p-region, no intervalley scattering happens (forbidden by the spin conservation). Upon reflection at the open boundary in the n-region, the pulse undergoes inter-valley scattering and becomes hybrid, and after which the component in Dirac valley and the component in quadratic valley show Klein and anti-Klein tunneling, respectively.

**Title: Supplementary Movie 6.**
**Description: Full wave simulation of a homogeneous nanoribbon of photonic quadratic-node semimetal for mode order 35.** The simulation setup is described in Supplementary Information Section 5. In the movie, the top two panels show the intensity distribution in the real and momentum spaces (with the same color scale as in Fig. 3) for the homogeneous nanoribbon excited on the left edge (with spin $(1,1)^T$), and the bottom panel shows the band structure ($k_y = 0$) with the black dashed line marking the excitation frequency.

**Title: Supplementary Movie 7.**
**Description: Full wave simulation of a homogeneous nanoribbon of photonic quadratic-node semimetal for mode order 34.** The simulation setup is described in Supplementary Information Section 5 and Supplementary Figure 12. In the movie, the top two panels show the intensity distribution in the real and momentum spaces (with the same color scale as in

Fig. 3) for the homogeneous nanoribbon excited on the left edge (with spin $(1,1)^T$), and the bottom panel shows the band structure ($k_y = 0$) with the black dashed line marking the excitation frequency.

**Title: Supplementary Movie 8.**

**Description: Full wave simulation of a photonic quadratic-node semimetal nanoribbon p-n junction.** The simulation setup is described in Supplementary Information Section 5 and Supplementary Figure 12, with the excitation set to spin $(1,1)^T$. In the movie, the top three panels show the intensity distribution in the real space and in the momentum space for n- and p-regions separately. The bottom panel show the band structure ($k_y = 0$) in the n- and p-regions, respectively, with the black dashed line marking the excitation frequency.
